# Impacts of environmental tobacco smoke on the onset and progression of multiple sclerosis: a systematic review

**DOI:** 10.1055/s-0044-1779271

**Published:** 2024-03-15

**Authors:** Marco Antônio Machado Schlindwein, Marcelo Henrique de Moura Campos, Leticia Caroline Breis, Beatriz Sordi Chara, Clara Sasse Scherer, Vitória Augusta Piva Caminski, André Matta, Marcus Vinicius Magno Gonçalves

**Affiliations:** 1 Universidade da Região de Joinville, Departamento de Medicina, Joinville SC, Brazil.; 2 Mayo Clinic, Department of Gastroenterology and Hepatology, Rochester, Minnesota, United States.; 3 No affiliation at the moment.

**Keywords:** Multiple Sclerosis, Disease Progression, Tobacco Smoke Pollution, Systematic Review, Esclerose Múltipla, Progressão da Doença, Poluição por Fumaça de Tabaco, Revisão Sistemática

## Abstract

**Background** Unlike cigarette smoking, environmental tobacco smoke (ETS) has not been as well described as an environmental risk for Multiple sclerosis (MS) nor as a risk factor for disease progression.

**Objective** We systematically reviewed the association between ETS and the risk of onset and/or progression of MS.

**Methods** We systematically screened MedLine/PubMed, Science Direct, LILACs, and SciELO searching for publications between January 1st, 2010, and July 5, 2021, with the following keywords: “multiple sclerosis and smoking”; “multiple sclerosis and passive smoking”; “multiple sclerosis and secondhand smoking”.

**Results** Fifteen articles were included in this review, which consisted of systematic reviews with meta-analysis (N = 2), systematic reviews (N = 2), and observational studies (N = 11). Both meta-analyses reported an impact of ETS on MS onset among secondhand smokers. One of the systematic reviews selected two observational studies showing the association between ETS and MS development, and one study that did not find a significant association between ETS and the risk of MS development. The other systematic review identified selected eight articles showing a relationship between ETS and MS. Seven observational studies reported higher odds of MS onset when associated with ETS. Four observational studies did not show a relationship between ETS and MS onset or progression.

**Conclusion** Most articles showed a positive association between ETS exposure and the risk of developing MS. On the other hand, an association between ETS and a higher risk for MS progression could not be established.

## Resumo

**Antecedentes** Ao contrário do tabagismo ativo, o fumo passivo (FP) não é tão bem estabelecido como risco para o desenvolvimento de esclerose múltipla (EM) nem como um fator de risco para a progressão da doença.

**Objetivo** Revisamos sistematicamente a associação entre FP e o risco de aparecimento e/ou progressão da EM.

**Métodos** Fizemos uma triagem sistemática nas bases de dados MedLine/PubMed, Science Direct, LILACs e SciELO em busca de publicações entre 1° de janeiro de 2010 e 5 de julho de 2021 com as seguintes palavras-chave: “multiple sclerosis and smoking”; “multiple sclerosis and passive smoking”; “multiple sclerosis and secondhand smoking”.

**Resultados** Quinze artigos foram incluídos nesta revisão, que consistiu em revisões sistemáticas com metanálise (N = 2), revisões sistemáticas (N = 2) e estudos observacionais (N = 11). As metanálises relataram um impacto do FP no surgimento da EM entre fumantes passivos. Um revisão sistemática selecionou dois estudos observacionais mostrando a associação entre FP e desenvolvimento de EM, e um estudo que não encontrou associação significativa entre FP e o risco de desenvolvimento de EM. Outra revisão sistemática identificou oito artigos selecionados mostrando uma relação entre FP e EM. Sete estudos observacionais relataram maiores chances de aparecimento de EM quando associados a FP. Quatro estudos observacionais não mostraram uma relação entre FP e o desenvolvimento ou progressão da EM.

**Conclusão** A maioria dos artigos mostrou uma associação positiva entre a exposição ao FP e o risco de desenvolver EM. Por outro lado, não foi possível estabelecer uma associação entre FP e maior risco de progressão da EM.

## INTRODUCTION

Multiple sclerosis (MS) is a chronic inflammatory demyelinating autoimmune disorder of the central nervous system (CNS). 1 It is the most common demyelinating disease in high-income countries, with a prevalence of approximately 140/100,000 inhabitants in North America and 108/100,000 in Europe. 2 The etiology of MS remains unclear, but environmental and lifestyle components, accompanied by genetic susceptibility, have been associated with an increased risk of MS. 1

Tobacco smoking has been consistently reported as an MS environmental risk, increasing the propensity of developing such a disorder. Moreover, smoking is additionally mentioned as a risk factor for a more aggressive disease progression. In a case-control study, smoking was associated with a 50% increase in risk for MS onset (odds ratio, OR 1.5, 95% confidence interval, CI 1.0–2.1). 3 Furthermore, a cross-sectional study showed that smoking after MS diagnosis is associated with a reduced time for the development of secondary progressive MS. 4

The mechanism underlying the relationship between smoking and MS is not entirely understood. In a study published in 2016, Okinger and colleagues showed that smoking increases the number of alveolar macrophages, in addition to altering the distribution of alveolar macrophages and lymphocytes in the bronchoalveolar lavage fluid. 5 With a proinflammatory environment, foreign antigens that are present in smoke, together with sequestered antigens, which are generated by mucosal cells injury, and neoantigens, which are promoted by reactive oxygen species, may result in a crossreaction with self-antigens, inducing autoimmunity. 6

Moreover, there seems to be an interaction between active smoking and human leukocyte antigen (HLA) complex genes associated with higher MS risk. A case-control study demonstrated the impact of HLA-DRB1*15 and HLA-A*02 on MS predisposition. When smoking was linked to the carriage of HLA-DRB1*15 and absence of HLA-A*02, the OR was 13.5 (95% CI 8.1– 22.6). On the other hand, smokers without both risk genotypes presented an OR of 1.4 (95% CI 0.9–2.1). 7

Unlike cigarette smoking, environmental tobacco smoke (ETS) – which is formed from the burning of cigarettes and other tobacco products and from smoke exhaled by the smoker – has not been as well described as an environmental risk for MS nor as a risk factor for disease progression. 8 It is important to note that although the overall exposure to ETS among non-smokers in the US has declined between the years 1988 and 2014, its prevalence has not decreased significantly in recent years. In addition, 25% of nonsmokers, including 14 million children, were exposed to ETS between 2013 and 2014. 9 Thus, it would be of great importance to establish whether ETS impacts the onset or progression of MS.

Therefore, we conducted a systematic review to analyze the association between ETS and the risk of onset and/or progression of MS.

## METHODS

This systematic review was conducted according to the recommendations of the Preferred Reporting Items for Systematic Reviews and Meta-Analyses (PRISMA) statement ( https://pubmed.ncbi.nlm.nih.gov/19621072/ ). There was no previous protocol registered.2.1 Literature search.

The authors systematically screened MedLine/PubMed, Science Direct, LILACs, and SciELO from July 5, 2021, to August 13, 2021, with the following keywords: “multiple sclerosis and smoking”; “multiple sclerosis and passive smoking”; “multiple sclerosis and secondhand smoking”. An 11-year filter was applied (from 2010 to 2021).

### Eligibility criteria

The eligibility criteria encompassed studies that:

• were published in English, Spanish or Portuguese;
• described the association between passive/secondhand/environmental tobacco smoking and MS;
• were published between January 1st , 2010, and July 5, 2021; and
• were either prospective or retrospective clinical trials, case reports, systematic reviews, or meta-analyses.

The present paper did not include studies that:

• presented other study designs, such as non-systematic reviews;
• were not indexed in the screened platforms; and
• did not describe the association between passive/secondhand smoke and MS. Additionally, duplicate articles were only counted once.

### Risk of bias

Two independent reviewers (CSS and VC) conducted the primary literature research using the previously described search terms. At first, titles and abstracts were screened based on the eligibility criteria. The full texts of all the screened abstracts were evaluated by a third reviewer (MAMS). In case of doubt, the most experienced author (MVMG) re-evaluated the studies. Two independent reviewers (BSC and MHMC) assessed the risk of bias in each systematic review and meta-analysis according to the AMSTAR 2 guidelines, 10 whereas observational studies were analyzed using The Newcastle-Ottawa Scale (NOS) for Assessing the Quality of Non-randomized Studies in Meta-analyses. 11 No study was excluded based on the risk of bias.

### Data extraction and synthesis

**Figure 1:**
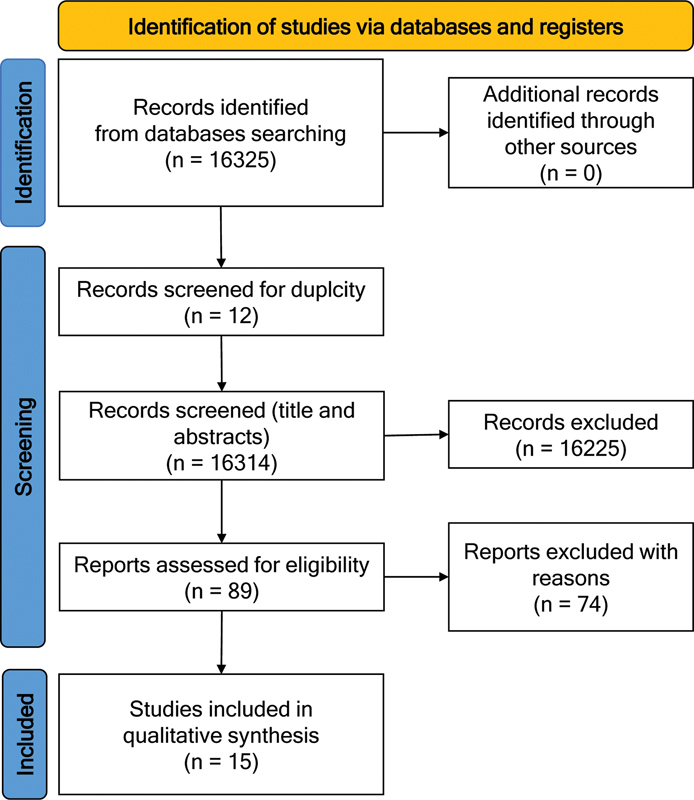
Flow chart, according to the PRISMA statement.

Using the keywords, 626 articles were identified in the MedLine/PubMed platform. In ScienceDirect, 15,696 articles were identified, and 12 of those were duplicated. Four articles were identified in LILACs. No articles were found on the SciELO platform. Thus, 16,314 articles were screened for title and abstract and 16,225 were excluded because they did not fit in the inclusion criteria, especially due to study design. Subsequently, 89 full texts were reviewed (83 from MedLine/PubMed and 5 from Science Direct) and 74 were excluded after full-text reading, mainly for not having data on ETS and MS. Finally, 15 studies were included in this review. Figure 1 illustrates the flow chart, according to the PRISMA statement.

## RESULTS

The 15 studies included in this review consisted of systematic reviews with meta-analyses (N = 2), systematic reviews (N = 2) and observational studies (N = 11). Among the 11 observational studies, four were exclusively about ETS, six were non-exclusively about ETS, and the last one was about waterpipe smoking. Table 1 summarizes all studies included with their populations, main findings, and limitations.

**Table 1:** Compilation of the included primary articles

Author	Study Design, country, and year	Population	Main findings	Study limitations
Poorolajal et al. 12	Meta-analysis, Iran, 2017.	−	The study indicated that both former and current smokers are predisposed to develop Multiple Sclerosis (MS). The risk increases proportionally to the number of cigarettes smoked per day. The meta-analysis selected 3 studies that show the association between MS and environmental tobacco smoke (ETS). Among second-hand smokers, the estimated Odds Ratio (OR) of MS was 1.12, when compared with nonsmokers.	(I) Does not identify which studies had the association between MS and ETS. (II) Data regarding environmental tobacco smokers was not detailly provided. (III) Does not detailly described selected studies. (IV) Limited number of studies in some subgroups. (V) The majority of articles selected were low-quality studies.
Zhang et al. 13	Meta-analysis, China, 2016.	−	The study shows that smoking is an environmental risk to MS. The meta-analysis also identified three studies containing four study populations that show the association between ETS and MS, reporting an overall OR of 1.24.	The study did not use a technique for assessing the risk of bias in the individual studies included.
McKay et al. 14	Systematic review, Canada, 2017.	−	Three studies selected evaluate the correlation between ETS and MS. Hedström et al. (2011), reported an OR of 1.3 for MS among never-smoker patients who were exposed to ETS. A French study, by Mikaeloff et al. (2007) reported an association between parental smoking and the early onset of MS in their children (OR 2.12), with a higher risk in older children, when compared to younger ones. Gardener et al. (2009) showed that children whose parents used to smoke at home had an increased risk of MS; however, when restricted to the cases that were non-smokers as adults, it was not statistically significant (OR 1.2, CI 0.90-1.5).	The vast literature present in the study limited the full discussion of some papers included, for example the strengths and limitations of each study.
Degelman and Herman. 15	Systematic review, Canada, 2017.	−	Four out of the eight articles analyzed, which cited the correlation between ETS and MS, demonstrated a statistically significant association. Meta-analysis was not performed due to heterogeneity among studies.	(I) Studies included regarding the association between ETS and MS were not described in detail. (II) Quality of study evidence for each outcome was either low or very low.
Oturai et al. 16	Case-control, Denmark, 2021.	N = 2,589. First cohort analyzed was composed by never-smokers (cigarettes), with 342 cases and 590 controls. The second was composed by individuals who started cigarette smoking above the age of 19, with 577 cases and 1,080 controls.	The association between exposure to ETS during adolescence (10-19 years of age) and MS was evaluated. In males, ETS exposure was not correlated to MS in neveractive smoking subjects. However, for those who became cigarette smokers in adulthood, previous ETS exposure history showed up with an OR of 1.593 for MS. ETS exposure in female neveractive smoking subjects demonstrated an OR of 1.43. There was no correlation between ETS in adolescence and MS in female patients that became active smokers after the age of 19 years.	(I) Selection of blood donors as controls. (II) Recall bias may be present, as subjects were asked about their experience and habits. (III) Authors considered ETS exposure only in the workplace or at home and did not consider the experience in other places, for example while outdoors.
Sakoda et al. 17	Case-control, Japan, 2020.	N = 227. Patients with MS: 103. Controls: 124.	MS patients were evaluated regarding environmental exposure risk factors. A history of exposure to ETS was observed to have a positive correlation with MS, with an OR of 1.31.	(I) Small sample size study. (II) Recall bias may be present, as subjects were asked about their past experience and habits. (III) Cases were hospital-based and at various clinical stages, potentially producing selection bias and heterogeneity between MS subjects (IV) Not possible to distinguish patients exposed to ETS as current smokers, ex-smokers, or never-smokers
Lavery et al. 18	Case-control, United States of America, 2019.	N = 297. All subjects aged less than 16 years; 216 children with monophasic acquired demyelinating syndromes (ADS); 81 children with MS.	The study concluded that ETS exposure was 37% more common in MS, in comparison to monophasic acquired demyelinating syndromes (29.5%); however, it was not an independent factor. When associated with the presence of HLADRB1*15, an OR of 3.7 for MS was reached.	(I) Recall bias may be present, as subjects were asked about their past experience and habits. (II) Authors considered ETS exposure only at home and did not consider the experience in other places, for example while outdoors.
Abdollahpour et al. 19	Case-control, Iran, 2017.	N = 1,604. Subjects with MS: 547; Controls: 1,057.	This article demonstrated that active waterpipe (OR 1.77), cigarette (OR 1.69) or secondhand (OR 1.85) tobacco smoking exposure is associated with increased MS risk. Regarding passive smoking, a much stronger association was found in exposures after the age of 20 years (OR∼2.2).	(I) Recall bias may be present, as subjects were asked about their past experience and habits. (II) Authors considered ETS exposure only at home and did not consider the experience in other places, for example while outdoors or workplace.
Hedström et al. 20	Case-control, Sweden, 2016.	N = 7,791. Among cases exposed to ETS: 457 never-smokers; 775 smokers. Among controls exposed to ETS: 1,115 never-smokers; 1,321 smokers.	The study assessed the impact of smoking and ETS on MS risk. The exposure to ETS in neversmokers was associated with the occurrence of MS in a dose-dependent manner, with an obtained OR of 1.1 for 1-20 years of exposure and 1.4 for more than 20 years of exposure	(I) Recall bias may be present, as subjects were asked about their experience and habits. (II) Authors considered ETS exposure only in the workplace or at home and did not consider the experience in other places, for example while outdoors.
Hedström et al. 21	Case-control, Sweden, 2011.	N = 2,330. Patients with MS: 695. Controls: 1,635. All subjects reported that they had never smoked before the year of MS onset.	This case-control study considered the exposure to ETS before the year of MS onset for cases and during the same period in the corresponding controls. Subjects who were exposed to ETS were found to be 30% more susceptible to develop MS. Furthermore, the exposure time was directly correlated with risk, when greater than or equal to 20 years, the obtained OR was 1.8, compared to individuals who had never been exposed.	(I) Recall bias may be present, as subjects were asked about their experience and habits. (II) Authors considered ETS exposure only in the workplace or at home and did not consider the experience in other places, for example while outdoors.
Hedström et al. 22	Case-control, Sweden, 2014.	N = 2,879. All subjects with MS. All subjects were never-smokers. Cases (never-smokers exposed to ETS): 1,311. Controls (never-smokers not exposed to ETS): 1,568.	This study evaluated the development of MS in groups of individuals who carried HLA-DRB1*15 and lacked HLA-A*02, which are genetic conditions that increase the susceptibility of MS (OR of 4.5). These patients presented a 7.7- fold higher chance of developing MS if exposed to ETS when compared to non-smokers never exposed to ETS without these HLA genotypes.	(I) Recall bias may be present, as subjects were asked about their experience and habits. (II) Authors considered ETS exposure only in the workplace or at home and did not consider the experience in other places, for example while outdoors.
Toro et al. 23	Cross-sectional, Colombia, 2020.	N = 174. Subjects with MS: 87. Subjects without MS: 87.	In the analysis, neither cigarette nor ETS history had a statistically significant association with an increased risk for MS.	(I) Recall bias may be present, as subjects were asked about their past experience and habits. (II) No detailed quantitative data on ETS amounts. (III) Subjects were asked about ETS exposure only considering when they were 19-25 years old
Abbasi et al. 24	Cross-sectional, Iran, 2016.	N = 660. All subjects with MS.	From the total of 660 patients with MS included in the study, most were female, with a median age of 37 years, and with relapsing-remitting MS clinical features. The analysis showed no association between ETS and MS severity.	(I) No detailed data on quantitative ETS amount. (II) Recall bias may be present, as subjects were asked about their experience and habits.
Mandia et al. 25	Cross-sectional, Italy, 2014.	N = 131. All subjects with MS.	The study examined factors that may be associated with the evolution of MS. There was no significant correlation between cigarette smoking status or exposure to ETS and the severity of MS.	(I) Small sample size study. (II) Recall bias may be present, as subjects were asked about their past experience and habits. (III) Not possible to distinguish patients exposed to ETS as current smokers, ex-smokers, or never-smokers. (IV) The study only considered the exposure to ETS 12 months prior to data collection.
Ramagopalan et al. 26	Case-control, Canada, 2013.	N = 3,913. MS cases: 3,157. Controls (spouses): 756.	MS cases and spouses were asked about cigarette smoking and ETS exposure. There was no correlation between ETS and MS in never-smoking patients. Exposure to ETS at home presented an OR of 0.87, whereas at the workplace the OR was 0.99.	(I) Recall bias may be present, as subjects were asked about their experience and habits. (II) No quantitative data on ETS amounts. (III) Small control sample, possibly underpowered to detect relevant effects. (IV) Authors considered ETS exposure only in the workplace or at home and did not consider the experience in other places, for example while outdoors. (V) The study did not collect information on paternal smoking.

Both meta-analyses included in the present study reported an impact of ETS on MS onset among secondhand smokers. One of them, published in 2017 by Poorolajal and colleagues, included three articles with an overall OR of 1.12 (95% CI: 0.87-1.36) and heterogeneity of I2 = 66%. 12 The other, published in 2016 by Zhang and colleagues, included 3 studies – of which 2 were also selected in the study by Poorolajal et al. – and showed an OR of 1.24 (95% CI 1.03-1.49) and a heterogeneity of I2 = 67%. 13

Furthermore, a systematic review from Canada selected 2 observational studies showing the association between ETS and MS development. 14 The first one was a French study showing a relationship between ETS and MS onset (OR of 2.12). 14 The other study is a cohort of female nurses from the USA, included in Degelman and Herman's article as well, and it did not show a significant association between ETS and the risk of MS development (OR 1.2, CI 0.90-1.5). 14 15 A third study, carried out by Hedström et al., was also included by McKay and colleagues and also selected for the present article, which will be further described. 14 21

The other systematic review identified for the present study selected eight articles showing a relationship between ETS and MS. 15 Four of them reported a positive association between ETS and MS development, whereas the other four studies showed no interaction. 15

Among the twelve observational studies identified, seven of them reported higher odds of MS onset when associated with ETS. A case-control study from Denmark showed an OR for MS risk of 1.43 among females in the first cohort studied, and an OR for MS risk of 1.593 among males in the second cohort. 16 The population included, comprising a total of 2,589 individuals, was divided into never-smokers, with 342 cases and 590 controls, and those who became smokers after the age of 19 years, with 577 cases and 1,080 controls. In men who became active smokers in adulthood, the association between ETS and the disorder showed an OR of 1.593 (95% CI 1.070- 2.372). In contrast, among women who had never smoked, ETS was associated with MS risk (OR 1.43, 95% CI 1.02 to 2.01). 16

Another study, from Japan, with a cohort comprising 227 individuals, reported that exposure to ETS was associated with greater MS risk, displaying an OR of 1.31 ( 95% CI 1.05-1.63). 17 Moreover, a cohort composed of 216 children with acquired demyelinating syndromes (ADS) and 81 children with MS showed that 37% of patients that progressed to MS were exposed to ETS, whereas among patients with monophasic ADS, 29.5% were exposed. 18 In Iran, a case-control study, with 547 subjects with MS and 1,057 controls, showed an OR of 1.85 (95% CI 1.48-2.32) for MS development. 19

Three studies from Sweden reported higher MS risk. One of them, a cohort with 7,791 individuals, displayed an OR of 1.1 (95% CI 1.0-1.3), another is a case-control study (695 cases and 1,635 controls) that showed an OR of 1.3 (95% CI 1.1-1.6), and the last one – associating ETS in a genetically predisposed cohort – revealed an OR of 7.7 (95% CI 5.5- 10.8). 20 21 22

Four observational studies did not show a relationship between ETS and MS risk. A cross-sectional study from Colombia, composed of 87 MS cases and 87 controls, all older than 18 years, reported that this association was not statically significant (p = 0.5959). 23 A study from Iran, composed of 660 individuals with MS, reported an OR of 0.79 for MS progression risk associated with ETS exposure. 24 The impact of ETS on MS progression was also analyzed in a study from Italy, composed of 131 individuals with MS, revealing that no significant relationship was found. 25 In a case-control study from the United Kingdom, comprising 3,157 MS cases and 756 controls, individuals that were exposed to ETS at home presented an OR of 0.87 (95% CI 0.71-1.41), whereas exposure at the workplace showed an OR of 0.99 (95% CI 0.71-1.41). 26

## DISCUSSION

### Systematic reviews and meta-analyses

The present paper included two systematic reviews and two meta-analyses. 12 13 14 15 However, none of them approached ETS exclusively. Three of them were focused on the effects of smoking and MS risk, including ETS among smoking types. 12 13 15 The other one included a variety of factors associated with MS development risk, relapse, and progression. 14

Among these papers, the most recent was published in 2017 by Degelman and Herman, who performed a systematic review utilizing the Bradford Hill criteria for causation between smoking and both MS development risk and MS progression risk. 15 There were eight articles on ETS and MS risk included in this study. Four of them showed positive results between the association of ETS exposure and the risk of MS; one was exclusively focused on children, one utilized cotinine as a marker of exposure, and the other two articles detected a dose-dependent risk using time of exposure as a measure. The other four studies included, however, failed to show a significant association between ETS and the risk of MS development. Three of them were case-control studies while the other one was a cohort of female nurses. In this systematic review, the authors were unable to perform a meta-analysis due to the heterogeneity among the eight studies. There were discrepancies regarding the definition of ETS, and the characteristics of the populations studied were not comparable. Degelman and Herman additionally commented on mixed results from other two meta-analyses, which were also included in the present paper and are discussed further separately. 12 13 15

One of the meta-analyses cited by Dengelman and Herman was published in 2017 by Poorolajal and colleagues, with a focus on the evaluation of different types of smoking exposure and their influence on MS onset. 12 Regarding ETS, this study shows an overall OR of 1.12 (95% CI: 0.87-1.36) and substantial heterogeneity of I2 = 66%. However, these results were briefly shown and were not described in detail in the meta-analyses. 12

The other meta-analysis was published in 2016. 13 Zhang and colleagues found a significant relationship between ETS and MS (OR of 1.24, 95% CI 1.03-1.49), but also a similar heterogeneity found in the study by Poorolajal and colleagues (I 2 = 67%). 13

The fourth systematic review included in the present study, published in 2017, was not focused on ETS and neither on smoking; instead, it encompassed all factors associated with MS onset, relapse, and progression. 14 McKay and colleagues included two articles on ETS that were published before 2010, which were not included in the present review due to the established publication year restriction. 14 The French study, that evaluated the risk of parental home smoking and MS development, shows a significant relationship between ETS in childhood and early MS onset (OR of 2.12). In addition, this study shows a higher risk in older children - older than 10 years - when compared to younger ones (relative risk, RR of 2.49). 14 The other study was the cohort of female nurses included in Degelman and Herman's article as well. 14 15 The third study, performed by Hedström et al., was also included by McKay and colleagues and also selected for the present article, which will be further discussed. 14 21

Hence, ETS was never the main topic of a systematic review. Even in papers published over the same period of time – with similar methodologies –, there is an important disparity in the results displayed. An important factor that may have caused this variety regards the large number of different terms representing ETS found in the databases (“passive smoking”; “secondhand smoking”; “smoking exposure”; “environmental smoking”). This can make it more difficult to select all articles of interest.

### Observational studies

There are eleven observational studies included in the present paper that analyzed the association between ETS and MS. 16 17 18 19 20 21 22 23 24 25 26 They are either case-control or cohort studies evaluating either ETS and MS development risk or the impact of ETS on MS progression. Four of these articles were designed exclusively to assess the relationship between ETS and MS, whereas the others assessed ETS among other different factors for MS development or progression.

Among the four studies designed exclusively to analyze ETS as an environmental risk factor for developing MS, the oldest one is a case-control study, published in 2011, that included only subjects that had never smoked, evaluating the impact of ETS on the risk of developing MS. 21 The authors also analyzed confounding factors such as serum vitamin D levels and Epstein Barr virus status in this population. The results showed that 39% of cases and 34% of controls were exposed to ETS and that the exposure occurred almost exclusively at home. The study found a higher MS risk in subjects exposed to ETS compared to those who had never been exposed. In addition, the article revealed a trend suggesting that longer exposure time resulted in higher risk, showing an OR of 1.8 (95% CI 1.2-2.6) with a 20-year exposure. 21

Another study by Hedström and colleagues included in our review provided data on the interaction between the HLA-DRB1 * 15 and HLA-A * 02 genotypes – the former being associated with an increased risk of MS and the latter having a protective effect for MS. 22 As in their previous study, confounding factors, such as vitamin D and Epstein Barr virus status, were analyzed and the selection of subjects was restricted to people who never smoked. Non-smokers who also have never been exposed to ETS presented a higher risk of developing MS when they presented both genetic risks (OR 4.5, 95% CI 3.3-6.1). When individuals with the same genotype were exposed to ETS, the risk of developing MS increased significantly (OR 7.7, 95% CI 5.5- 10.8), showing that ETS may be an independent risk factor for MS onset. 22

Lavery and colleagues explored the effects of ETS in a USA cohort composed of children with ADS and children with MS. 18 The study showed that both groups had an association with ETS exposure. In addition, an investigation between the interaction of HLA-DRB1*15 and ETS was carried out, showing that carriage of this genotype – which was observed in 41.9% of the MS cohort – without ETS exposure did not increase the risk of developing MS. When both ETS exposure and the genotype were present, the OR for MS increased to 3.71 (95% CI 1.17-11.9). Together, these two factors accounted for a 54% MS risk. 18

The most recent study that focused on ETS as a risk factor for developing MS was published in 2021. 16 Oturai and colleagues explored the exposure to ETS during adolescence (from 10 to 19 years old) and its relationship with MS. Subjects that were smokers before the age of 19 years were excluded. Interestingly, ETS was not associated with MS in men who had never smoked. However, in men who became active smokers in adulthood, the association between ETS and the disorder showed an OR of 1.593. On the contrary, among women who had never smoked, ETS was associated with MS risk, increasing the odds of developing the disorder by 4.6% for each year of exposure. Also, in women who became active smokers after 19 years of age, ETS during adolescence did not increase the chances of developing MS. 16

Regarding the remaining studies included in this article, none of them were designed exclusively to assess the impact of exposure to ETS on MS risk. 17 19 20 23 24 25 26 Among these studies, two of them – one from Japan and the other from Sweden – showed that ETS was associated with an increased risk for MS. 17 20 In the Japanese study, ETS exposure was evaluated in subjects older than 16 years, showing a positive relationship with MS. 17 In the Swedish cohort, individuals who were exposed to ETS for more than 20 years and reported actively smoking more than 10 pack-years had an almost three-times higher risk of developing MS. Interestingly, this cohort also showed a dose-response relationship between years of exposure to ETS and the risk of MS. Individuals who were exposed to ETS for less than 20 years displayed an OR of 1.1 (95% CI 1.0-1.3) for MS, while exposure for more than 20 years showed an OR of 1.4 (CI 95% 1.1-1.8). 20

With regards to studies that found negative results, a Canadian case-control study performed a questionnaire to investigate lifetime cigarette smoking and ETS exposure with MS development. 26 Among never-smoker subjects (N = 1,394) there was no association between MS risk and ETS exposure either at home or at the workplace. 26 The other is a Colombian study in which subjects were interviewed to assess environmental risk factors for MS. Regarding ETS, subjects were asked if they were exposed between the ages of 19 to 25 years. Neither cigarette nor ETS history had a statistically significant association with an increased risk for MS onset. 23

Two additional studies evaluated the impact of ETS on the disease's severity and progression; however, they showed no association. 24 25 An Italian cohort completed a questionnaire about lifetime smoking habits. Moreover, the authors of the study considered ETS exposure only over the 12 months prior to the questionnaire. The study did not identify a significant association between either cigarette smoking status or exposure to ETS and the severity of MS. 25 In the other study, an interview regarding environmental risk factors for MS severity was carried out with an Iranian cohort. This study showed that ETS was not related to MS severity. 24

The last one, an Iranian case-control study evaluated the risk between waterpipe smoke and MS development. 19 This article showed that, besides active waterpipe smoke, ETS was associated with MS risk, displaying an important impact with a dose-dependent increase in risk. Interestingly, in this study, ETS during childhood and adolescence was not statistically significant for increasing the risk of MS. Furthermore, the study shows that subjects exposed to all three modes analyzed (waterpipe smoke, tobacco smoke, and secondhand smoke) had a 4.1 higher odds of having MS compared to subjects that were not exposed. 19

As with the systematic reviews and meta-analysis, the majority of observational studies also did not focus on ETS exclusively. In addition, there may be imprecision in the methods of quantifying exposure to ETS. Unlike active smoking, which has some quantitative measures such as pack years, exposure to ETS is usually measured based on questionnaires referring to the subject's exposure history, which makes the information more susceptible to recall bias.

### Study limitations

As a limitation of our study, the large number of different terms representing ETS found in the databases may have led to difficulties in selecting all articles of interest; With that in mind, the researchers examined a large number of studies, including all different terms that could represent ETS.

In conclusion, as shown from the studies selected by the present review, the majority of articles displayed a positive association between ETS exposure and the risk of developing MS. In addition, when considering only studies that were exclusively designed to evaluate ETS exposure, the association was found in all of them. Some articles showed that exposure during childhood resulted in a considerably higher risk. Also, the reviewed data about the relationship between ETS and genetic risk factors, such as HLA-DRB1 * 15 and HLA-A * 02 genotypes, shows a strong impact on the odds of developing the disorder. Moreover, a dose-dependent risk relationship between years of exposure to ETS and MS risk was observed in some cohorts. On the other hand, the association between ETS and a higher risk for MS progression could not be established.

Overall, the present study shows evidence of the impact of ETS on the development of MS and encourages further studies addressing this topic to strengthen the establishment of this association.
